# Supplementary material for: AtCHE1, the Arabidopsis homolog of mammalian AATF/Che-1 protein, is involved in safeguarding genome stability
Source: Commun Biol. 2025 Sep 2;8:1329. doi: 10.1038/s42003-025-08490-1 (PMC12402150; doi:10.1038/s42003-025-08490-1)
Supplement: Supplementary file 2 — Supplementary Information [file 42003_2025_8490_MOESM2_ESM.pdf]

Supplementary Table 1. Primers used for genotyping

Primer names	Sequence (from 5' to 3')
atr2: (SALK_032841)	
SALK_032841_LP	GCAGCAAAAATTTCTTGGTTG
SALK_032841_RP	ACTTCAAGGGTTCCGATGTTC
LB	GCGTGGACCGCTTGCTGCAACT
sog1-1	
sog1 Forward	CTATGTTGTTGTTGTAAACACAA
sog1 Reverse	CCAAATGGTATTGGTGCATCACCCAGTTGG
che1	
che1 mutant F	TAAGCTATGCTCTTCTCCTCTTC
che1 mutant R	CCTCTGGATTTGGTTCCAT

Supplementary Table 2. Primers used for mapping

Markers	Name	Sequence from 5' to 3'
MSN2	457122_F1:	TAAATGGGCGAGACAACATCACAG
	457122_F2:	CTATAGTGGGACTGAAAAGCCC
	457122_R1:	GGAAGTTGGTGAAAGAGCGGC
	457122_R2:	GTAGCAAACTCATCGCGTTGGGG
MUB3	457274_F1:	GGGCATAGAATAAGAGCATATTCC
	457274_F2:	GAATAAGGCAGTGGCAACCATTG
	457274_R1:	GAGAATACAATATTCGTGAGAG
	457274_R2:	CCATCACTCAAATTTGAACGTTGC
K11J9	454021_F1:	CGTTTTACGAGCATGGTCTTGGC
	454021_F2:	CATGGTCTTGGCTAACATCCTCC
	454021_R1:	CCAAACTCCTCGTGTTTGGCTG
	454021_R2:	TGGCTGACCGGTTATGATGAGG
F15L12	449419_F1:	CCTGACTATCCTCACTTCTGAGG
	449419_F2:	TTGAGGTTCATGTTTCTATGACG
	449419_R1:	GCCAAAGGGTAAGTGGGTGGGTGA
	449419_R2:	CAGAGACAGAGACGGTGAGTTAGAC
MTE17	457148_F1:	GGGTTCACGTGAACATGACAC
	457148_F2:	GATCGATCGATAGGAAGTGTTGG
	457148_R1:	CTGAAGCTGTGTTGCTCGTTGAG
	457148_R2:	GCGGGGTGATGGAGAGATTCAC
MTH12	MTH12_F:	CGGCATCTGTTCATGCATTATA
	MTH12_R:	CTCAAGGCTAAGTAGTGTATGA
MMN10	MMN10_F:	CCCCATTGCCCCGCGGTAATAAGC
	MMN10_R:	CGAACCATCACCACTGGTGAGTG
F15L12	F15L12_F:	AGTAGGAATTGGAATGAGCAA
	F15L12_R:	TGTCATGTGAGGACAAGTCTGAA
MUP24	MUP24_F:	GTAGAATCAGAAATACCATAATC
	MUP24_R:	GGTGTCCAATCAAGTTTTCGGTT
MAE1	MAE1_F:	GAACTGGACTATAATATAAATT
	MAE1_R:	GACTTGGGTCAGAGGACAAACG
MSL3	MSL3_F:	CTTACGTTTGTGAACACATATTA
	MSL3_R:	GACAAAATTGCAAACGCGGAGAT
MFB13	MFB13_F:	GACGACTGATTACATAACATAGT

	MFB13_R1:	GTGTGTATTGTCTATATATATTACG
	MFB13_R2:	AGCTGTCATGCGTGATGCTTG
MAC9	MAC9_F:	CACATGAGACTTGAGTGTTGTTC
	MAC9_R:	CGGATTTCATACGAACTTGCTAA
K19B1	K19B1_F:	GGTTATCGAATATATAAAATGTG
	K19B1_R:	GTCAATGTTGGGAATTTGCAGTCC
MQB2	MQB2_F:	GGCGACTACTAGCATAAAAAATA
	MQB2_R:	GATCTTGCCATTTATTTGGTCAA
MBK5	MBK5_F:	GGCCCATCTAGAGTATAACCATG
	MBK5_R:	CAGCTACTGCGTGCAAATAAAGAT
MGI19	MGI19_F:	CTAGAGAGACAAGATAAGACACC
	MGI19_R:	GTTATCGCCAAACTTGACCCTTA
MUB3	MUB3_F:	GCCTGGCTGATATTATGAACTTTC
	MUB3_R:	GGCTATTATCACTTCCGAAGAGGTT
MUB3	457274_F3:	GCATTGAATAAGGCAGTGGCAACC
	457274_R3:	CGTGTGGCAAATCCAATAGTTAG
MAF19	MAF19_a_F:	CTCAACCAATCAAAGGCGGACACC
	MAF19_a_R:	TATTAAACGATAAATTCGCCGTTTGC
MAF19	MAF19_b_F:	GTCACTGTTGTCTTTCTAGAAACAGAG
	MAF19_b_R:	CTCTATCTCTCTCTGTGTCTCTCCA
10A10	10A10_F:	GGTCACAGGGATCAAGATGTGG
	10A10_R:	GCCTTATGGATTTTCTGGAGAAAG
EG7F2	EG7F2_F:	GCATAGAATTTGACGATAACGAGC
	EG7F2_R:	GATCTGTGTAGGACTACGAGAC

Supplementary Table 3. The sequence information for the primers used for mapping

Name	Sequence from 5' to 3'
210_F1:	GGGTCGTTCTGAGTCGTCTCC
210_R1:	GTCTCTCAGGATAATATCAC
210_F2:	GTAGTAACCAATCCAAGTGTTCC
210_R2:	CTCACACCAACTAATGTCCTCAG
210_F3:	GCCGTTGAAATCGACCATGATC
210_R3:	CTCCACTCTCTGATAAGCATC
210_F4:	GGGACTGAACTCAGCACCCAGAG
210_R4:	ACTGGAAAGATTCTTATCATG
200_F1:	GGTAATGGAGAGATTCTCATG
200_R1:	CTGGTTTATACAGCAGGAAGAGGAAC
190_F1:	TCGACGAGATAGTGAGAGGAGTA
190_R1:	ATTCCCCTGTGGCTCCACTGC
190_F2:	CCAGAGAAAAGCATGTTCCAAGAGG
190_R2:	CACTTGCATTACGCAGTGCAGTCA
190_F3:	GATATTATAGCTTTGGATTAGGTAC
190_R3:	GACAAAGTTTAGATGCAGCTGGTC
190_F4:	GGAGAAACTAAGCATTCATGGGAGG
190_R4:	GATCGGTTTCTTCTCGGATTATCCTTG
190_F5:	CGAGGGGCACCAGAAGATAAAGTGG
190_R5:	CATCCATTGTTGACAACTTAAACATG
180_F1:	ACAATCTCTAAACCCTGACCTCCC
180_R1:	CGATTAGGGGGTCCTGCAGATGCGC
170_F1:	AGTATCGCCGACGCCGCAGCACA
170_R1:	GGGAATTCTACTACTGGTTTTCATC
160_F1:	TTAACTCCATCTGATACTCAGCTG

160_R1:	GGGTCACCACTACGTGCCTTCACCATG
160_F2:	CACGCGCTGAGCTTCACTTGTCAA
160_R2:	AGGGGGAAATAGATTCACTGATCATG
150_F1:	CGTCGGAGCTCAGATCACGAGCCG
150_F1:	GTTCATCCTGCTCAACGTCATTCCGAAC
150_F2:	GAGGTTCAAGTTGCACAGTCAGACG
150_R2:	GCATCAGAAGTACTAGTAATAGGC
150_F3:	ACATATGTAAGTGAACACTTCTTC
150_R3:	CATCAAGAGCATCTTCCAAGTAGCC
150_F4:	ACAAAGCTGAGTCAGGCGAGGGAG
150_R4:	AACGAGTCTAATTCCAGACTACAT

Supplementary Table 4. Primers for vector construction

Primer names	Sequence (from 5' to 3')
Promoter reporter analysis with GUS	
Forw: 164-6:	GGGGCGATCGGCGGCCGCGGAGACAGAGATATCATATCCATAGCTC
Rev: 164-7:	GGGGGGCGCGCCAAATCAGGTAATTGATCATCTTCATTGTCGCT
Complementation analysis of mutant plant lines	
Forw: 164-35:	GGGGGCCTGCAGGGGAGACAGAGATATCATATCCATAGCTC
Rev: 164-11:	GGGGGAGCTCGTGAAAGAGACGCAGAAGATGTGAAACC
Subcellular localization	
Forw: 164-35:	GGGGGCCTGCAGGGGAGACAGAGATATCATATCCATAGCTC
Rev: 164-12:	GGGGGGCGCGCCAAGCTTCAGACTGAACGTTTCTGGTCTTG

Supplementary Table 5. Primers for qRT-PCR

Gene names	Forward Primers (from 5' to 3')	Revers Primers (from 5' to 3')
PLT1	TTGCAGCAACAGTCGAGCCAGA	TCGGTCGATCCAACAGTCGAGC
PLT2	GGCTGAGGAAGAGTTTCCAGCCG	TCCATACCCTACCTTGCCTCGC
BRCA1	TCATGGGAGATTTCGAGCTT	ATTTAGCCAAGGCTTCAGCA
RAD51	TTGCTGGTCCCCAATTTAAG	CAAACATGGCGAGCTTATCA
SCR	AGCAACAACCGTGGTCCTCCT	ATACAGTAGCCGCCGCCGTAA
SHR	GCGAACGATGCTACCGAACCAT	GCCTCTCCGTCTACTGCTTCCA
PARP2	ATGGCGTTCTGCTCCTCTGC	GGTGCTGTTTTCCCCACACC
AtCHE1	GAAGAATCAGAAGGCTCTTTGGG	CTCAAACAAAGCCTCTTGCAAC
UBQ10	CCTGCGTCTTCGTGGTGGTT	GTCGAGTCACTTTGCAGGCGT

Supplementary Figure 1

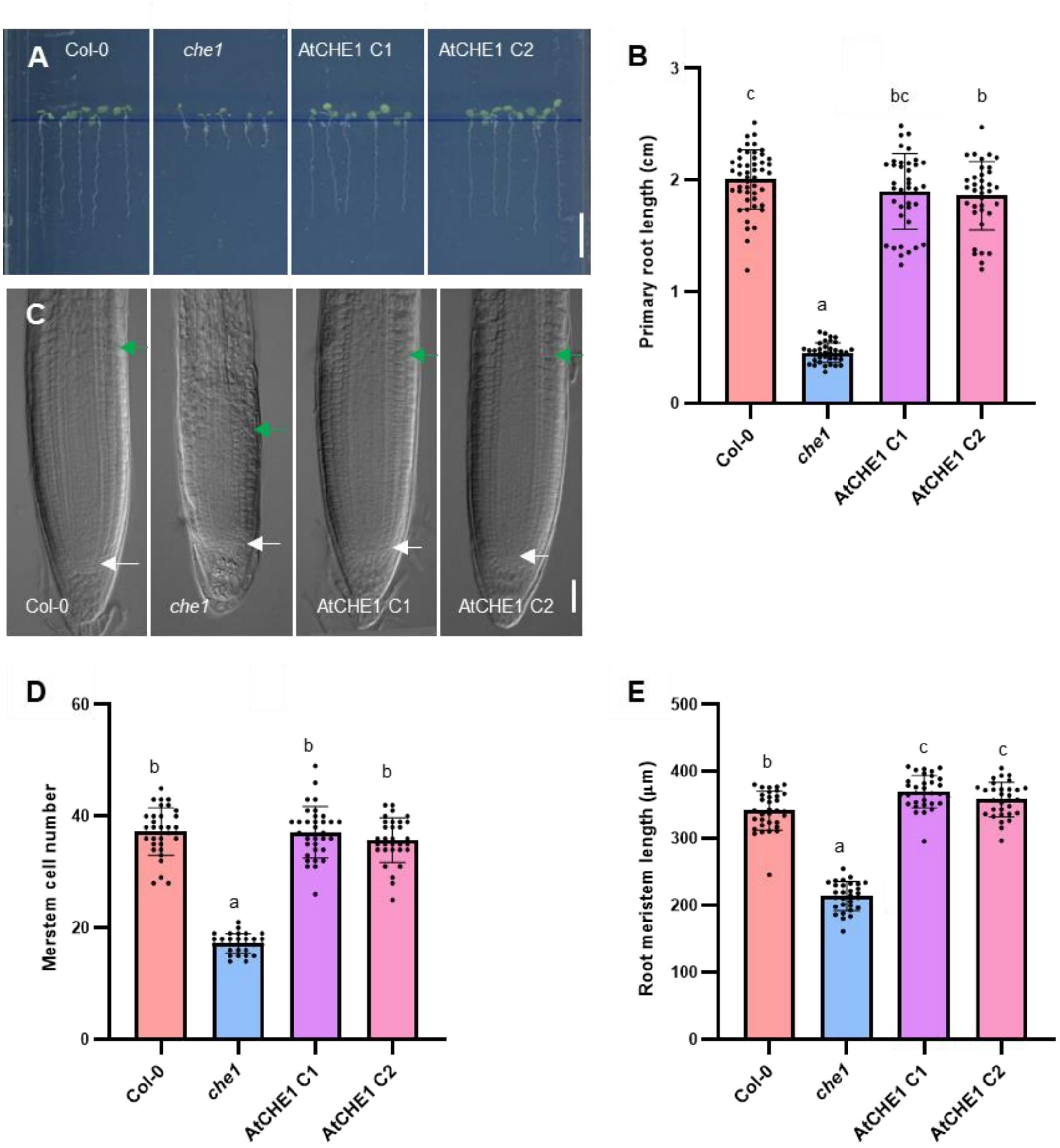

Supplementary Figure 1. *AtCHE1* genomic DNA fragment can complement the *che1* phenotype. (**A**) Complementation of the *che1* mutation with the *AtCHE1* genomic DNA region expressed under its own promoter. Root growth phenotype of Col-0, *che1* and the complementation lines $AtCHE1_{pro}$-$AtCHE1_{g}$-$AtCHE1_{3'\text{-}UTR}$ (AtCHE1 C1 and AtCHE1 C2, 6 DAG). Scale bar = 1 cm. (**B**) Primary root length (6 DAG, cm). (**C**) Root meristem of Col-0, *che1* and complementation lines. Scale bar = 50 µm; white arrowheads point to QC, while the green one to the first elongated cortical cell. (**D**) Number of the amplifying cell in the cortex cell line and (**E**) Root meristem length. The value and error bars in (**B**), (**D**) and (**E**) represent means and ±SD, n>20 in each. Columns with different letters indicate significant differences at $P<0.05$ (Duncan's multiple range means comparisons).

Supplementary Figure 2

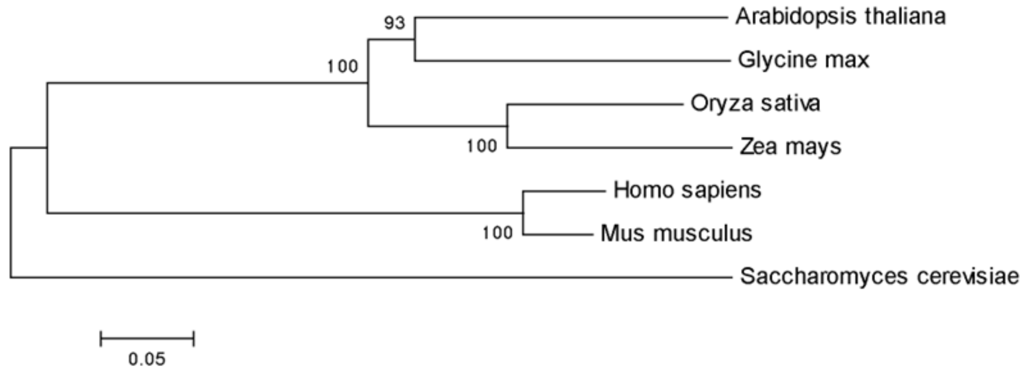

Supplementary Figure 2. AtCHE1 is the plant homolog of AATF/Che-1.

Phylogenetic analysis of AATF/Che-1 from different species. NCBI blast, Mega 5.05, align by clustal W, construct/test neighbor joining tree. Bootstrap test using 1000 repetitions.

Supplementary Figure 3

```
Saccharomyces    --meksladqisdi-aikpvnkdfdi--edeenaslfqhnekngesdlsdygnsnteetk   55
Homo             magpqplalqleqllnprpseadpeadpeeataarvidrf-dege-------------dg   46
Mus              maapqplalqleqllnprpreadpeadpeeatrarvidrf-dege-------------ee   46
Arabidopsis      ------------------------------------------------------------   0
Glycine          ------------------------------------------------------------   0
Oryza            ------------------------------------------------------------   0
Zea              ------------------------------------------------------------   0

Saccharomyces    kahylevekskl raekglelndpkytgvkgsrqalyee----ase---------------   96
Homo             egdflvvgsirklasaslldtdkrycgkttsrkawnedhweqtlpgssdeei--------   98
Mus              kdd-lavssirklapvslldtdkrysgkttsrkawkedhweqalpsssdnea--------   97
Arabidopsis      -mrlsialkvvkmaggs-----------krskrarlds----esedisdqenlkaesdne   44
Glycine          ----------------------------------------------------m---glsa   5
Oryza            ------------mapgt-----------tlapkrrkae----aspsp-spspm---gdss   29
Zea              ------------------------------------------------------------   0

Saccharomyces    ---------nedee------eeeeeeeegeeeeeeegeeeee------------------   123
Homo             --sdeegsgdedseglgleeyd--eddlgaaeeqecgdhreskksrshsaktpgfsvqsi   154
Mus              --sdeggsedgdseglgleeis--edvdedlednkisde---------------------   132
Arabidopsis      ddqlpdgieddevdsmeddegeseeddegdteeddegdseed------------------   86
Glycine          kksrkrgkrdsdsdeydnmeye------------eveddyd-------------------   34
Oryza            d----ggysdsdlhdaeesfysar---sgseddrqvsssndd------------------   64
Zea              ------------------------------------------------------------   0

Saccharomyces    -----------------eeekeed-alsfrtdsedeeveideeesdadggeteeaqqkrh   165
Homo             sdfekftkgmddlgsseeeedeesgmee--gdda-edsqgese--edrag----------   199
Mus              -------------ggsedgdseglgleefsedve-edlegede--edree----------   166
Arabidopsis      -------------degenkededgesedfedgndkesesgdegnddnkdaqme---elek   130
Glycine          -------------ddgeeed-e----eeehgeevtddedgtgehgewkndeme---qlek   73
Oryza            -------------ddseeeeqeeremdeeedeeddddeemneeddedegeemn---elek   108
Zea              ------------------------------------------------------------   0
```

Continue of Supplementary Figure 3

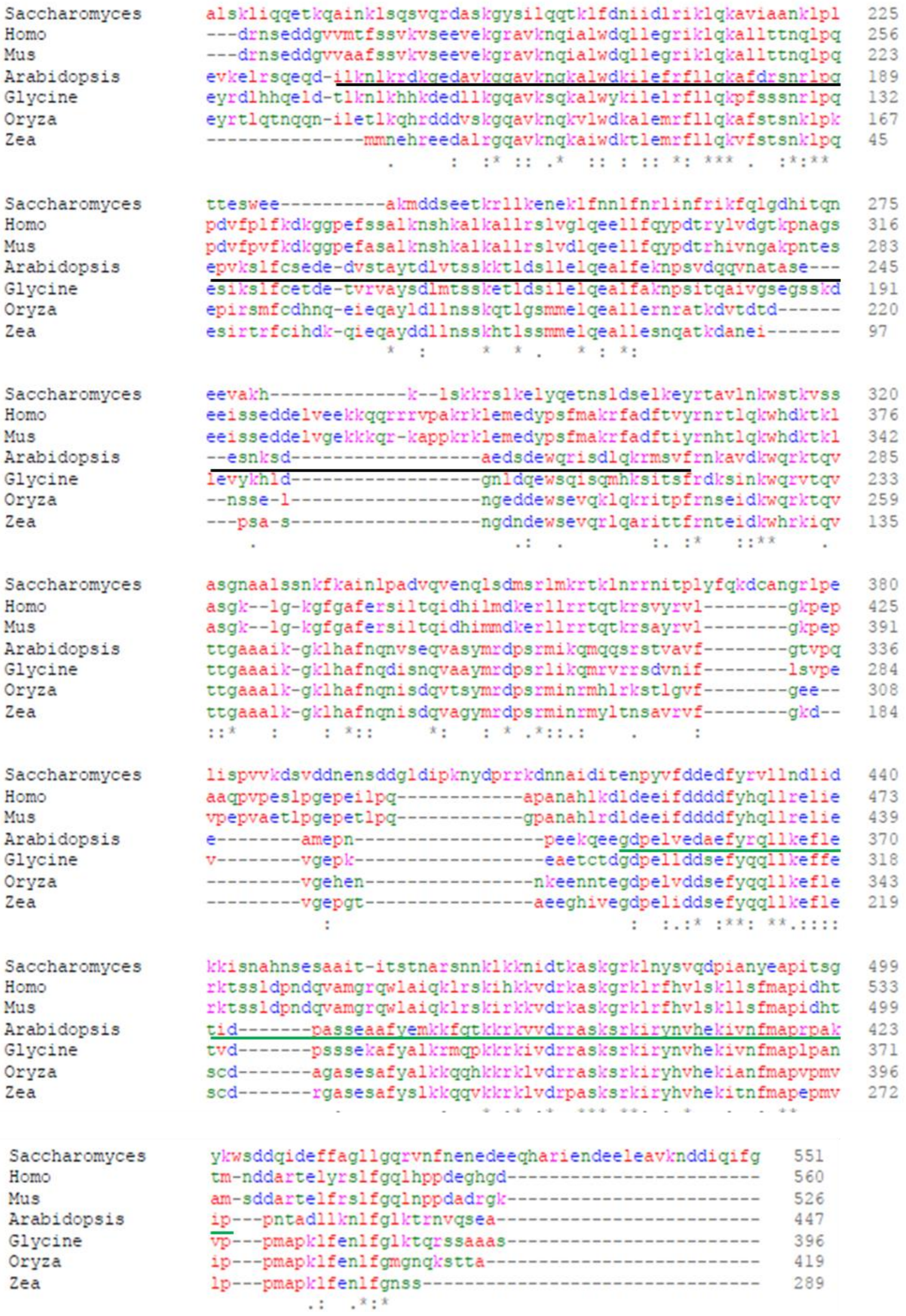

Supplementary Figure 3. The alignment of the AATF/Che-1 protein sequences

Alignment of AATF/Che-1 via clustalw2 (http://www.ebi.ac.uk/Tools/msa/clustalw2/). Black line indicates AATF domain and green line indicates TRAUB domain of Arabidopsis.

Supplementary Figure 4

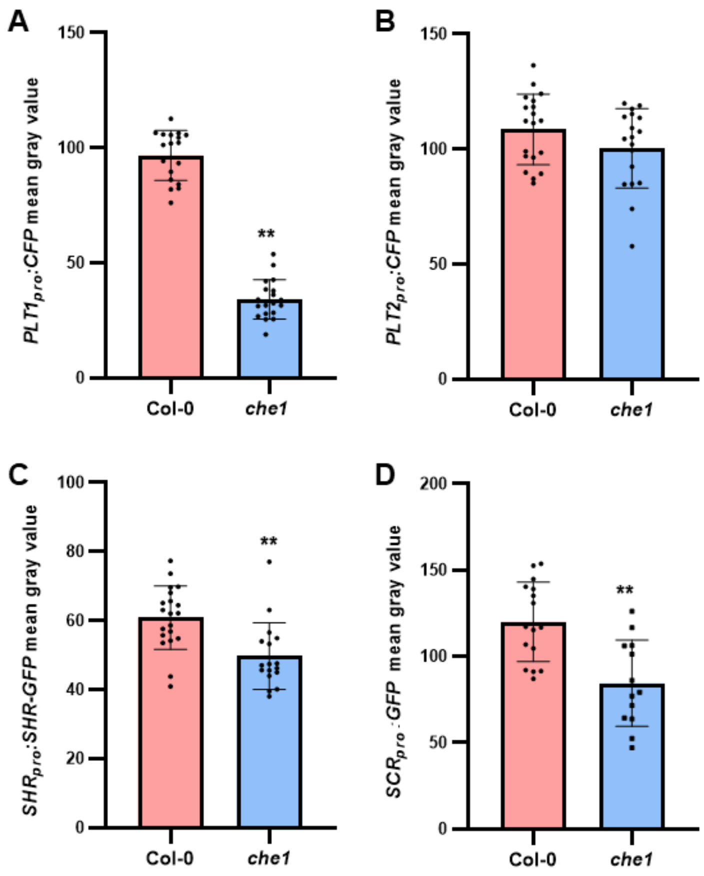

Supplementary Figure 4. Expression levels of *PLT1*, *PLT2*, *SHR* and *SCR* are reduced in *che1* compared to Col-0.

Mean gray value of *PLT1*$_{pro}$*:CFP* (**A**), *PLT2*$_{pro}$*:CFP* (**B**) *SHR*$_{pro}$*:SHR-GFP* (**C**) and *SCR*$_{pro}$*:GFP* (**D**) in Col-0 and *che1*. The values and error bars in (**A**) - (**D**) represent means and ±SD, n>10. Asterisks indicate significant differences compared to Col-0. (***P* value < 0.01, two-tailed Student' s t test, n>10).

Supplementary Figure 5

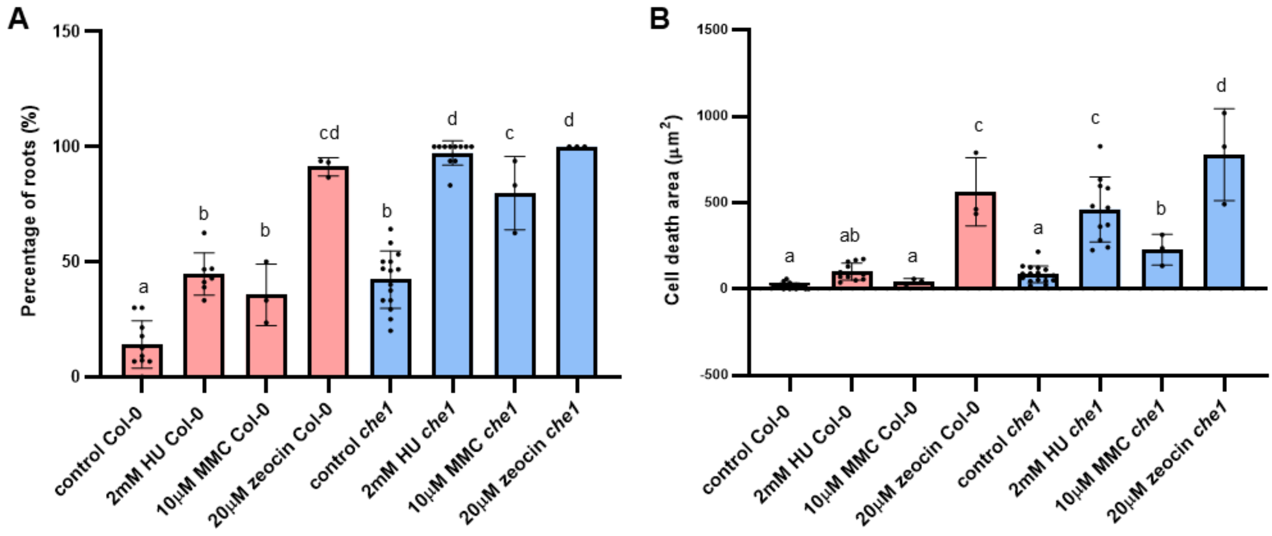

Supplementary Figure 5. Response to DNA damage treats in *che1* compared to Col-0.
(**A**) The proportion of roots with cell death and (**B**) the mean area of dead cells after treatment compared to the control. Data in (**A**) and (**B**) are the means and ±SD from at least three independent experiments with at least 30 seedlings in total. Columns with different letters indicate significant differences, *P*<0.05 (Duncan's multiple range means comparisons).

Supplementary Figure 6

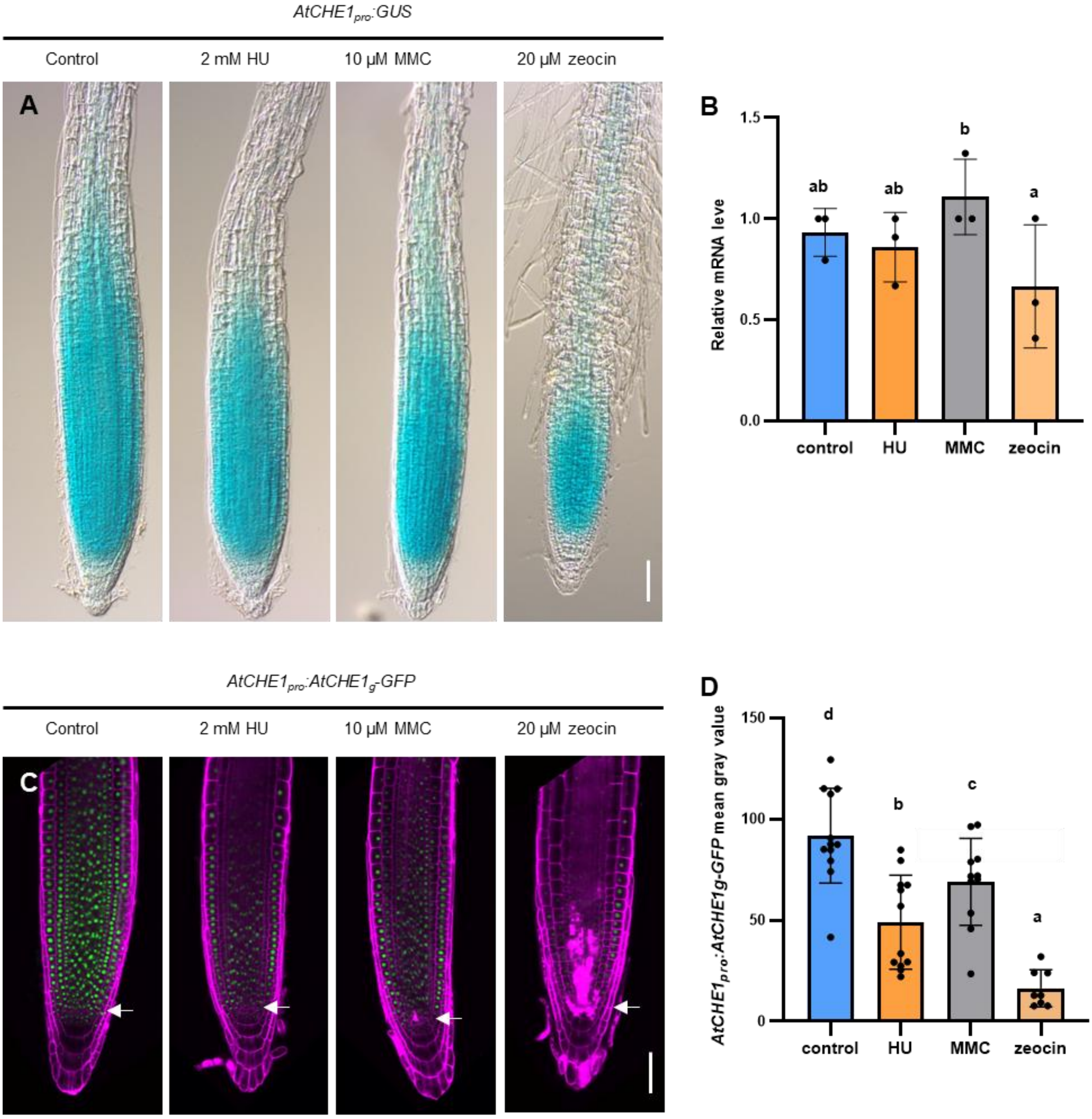

Supplementary Figure 6. *AtCHE1* fusion protein level is affected by DNA damage reagent treatment.

(**A**) GUS-stained images of *AtCHE1* promoter fused to *GUS* reporter gene (*$AtCHE1_{pro}$:GUS*) seedlings. 5 DAG seedlings were transferred and grown for 1 day on 1/2 MS medium with either no stress, 2 mM HU, 10 µM MMC, or 20 µM zeocin respectively. Scale bar = 50 µm. (**B**) Relative mRNA levels (gene vs reference gene) of *AtCHE1* under DNA damage stress treatments were measured by qRT-PCR. 4 DAG seedlings were transferred to 1/2 MS liquid medium without stress, 2 mM HU, 10 µM MMC, or 20 µM zeocin and grown for 24 h. After that, 5 DAG seedlings were collected as samples. The values and error bars represent mean and ±SD from three independent experiments. Columns with different

letters are significantly difference at P<0.05 (Duncan's multiple range means comparisons). (**C**) Confocal images of PI-stained of *AtCHE1*$_{pro}$*:AtCHE1*$_{g}$*-GFP* root tips. 5 DAG seedlings were transferred to 1/2 MS plates with no stress, 2 mM HU, 10 μM MMC or 20 μM zeocin and grown for 24 h. Magenta, PI staining and green, GFP. Scale bar = 50 μm; arrowheads point to QC. (**D**) Gray value of *AtCHE1*$_{pro}$*:AtCHE1*$_{g}$*-GFP* under DNA damage treatment. The values and error bars represent mean and ±SD, n>8. Columns with different letters are significantly difference at P<0.05 (Duncan's multiple range means comparisons).

Supplementary Figure 7

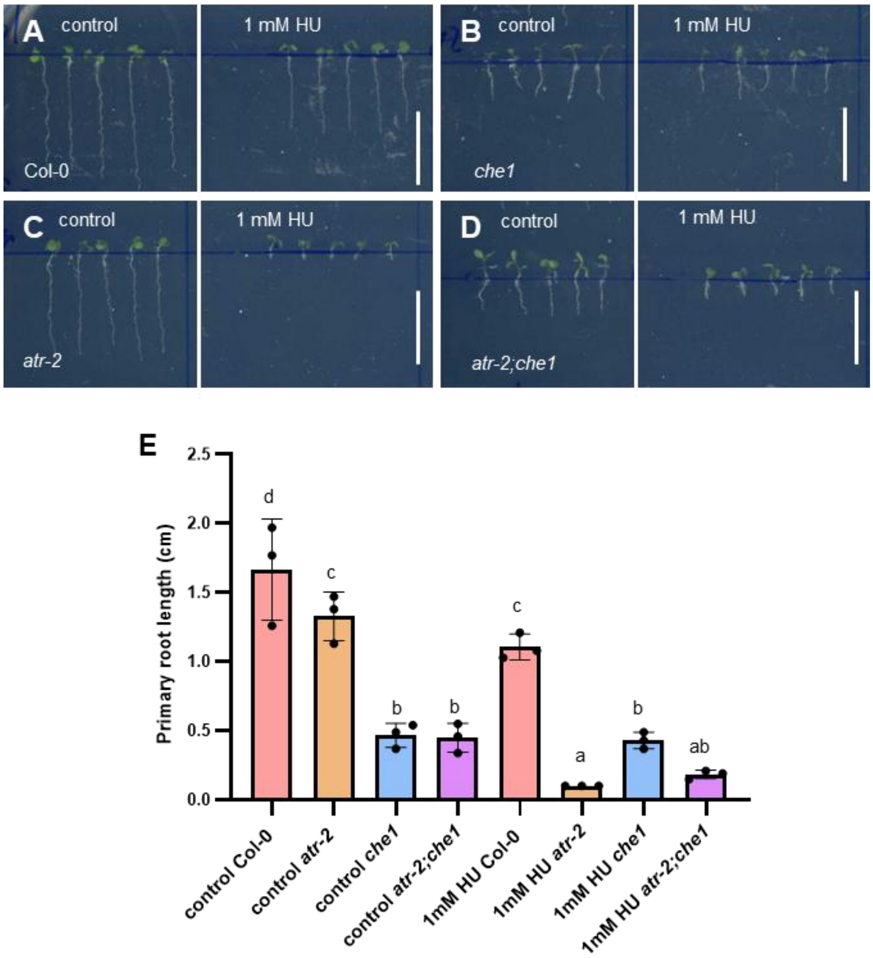

Supplementary Figure 7. Primary root length of *atr-2;che1* double mutant is partially rescued compared to *atr-2* mutant upon HU treatment.

(**A**) to (**D**) Primary root phenotype of 6 DAG Col-0 (**A**), *che1* (**B**), *atr-2* (**C**) and *atr-2;che1* (**D**) seedlings grown on 1/2 MS medium containing 0 (control condition) or 1 mM HU. Scale bars = 1 cm. (**E**) Measurement of primary root length of Col-0, *che1*, *atr-2* and *atr-2;che1* (6 DAG). The value and error bars represent the means and ±SD from three independent experiments with at least 10 seedlings each. Columns with different letters are significantly differences at $P<0.05$ (Duncan's multiple range means comparisons).
