# Supplementary material for: AtCHE1, the Arabidopsis homolog of mammalian AATF/Che-1 protein, is involved in safeguarding genome stability
Source: Commun Biol. 2025 Sep 2;8:1329. doi: 10.1038/s42003-025-08490-1 (PMC12402150; doi:10.1038/s42003-025-08490-1)
Supplement: Supplementary file 3 — Description of Additional Supplementary Files [file 42003_2025_8490_MOESM3_ESM.pdf]

Description of Additional Supplementary Files

File name: Supplement data 1

Description: Genomic and CDS sequence of wild type AtCHE1 and che1 mutant

File name: Supplement data 2

Description: The numerical source data for the graphs and charts
