# Supplementary material for: AtCHE1, the Arabidopsis homolog of mammalian AATF/Che-1 protein, is involved in safeguarding genome stability
Source: Commun Biol. 2025 Sep 2;8:1329. doi: 10.1038/s42003-025-08490-1 (PMC12402150; doi:10.1038/s42003-025-08490-1)
Supplement: Supplementary file 4 — Supplementary Data 1 [file 42003_2025_8490_MOESM4_ESM.docx]

**Supplement data 1 genomic and CDS sequence of wild type *AtCHE1* and *che1* mutant**

*AtCHE1* (AT5G61330) genomic sequence from ATG to TGA:

ATGGCTGGGGGGTCAAAGAGGTCTAAAAGAGCAAGACTTGACAGTGAATCGGAAGACATAAGCGACCAAGAAAACCTTAAGgtatgtttatgttgcttttgtctcgtttaagttatttagatggtataggtgaatttatttggttgagggttctctatattatagGCCGAAAGCGACAATGAAGATGATCAATTACCTGATGGGATAGAGGATGATGAAGTAGATAGCATGGAAGATGATGAGGGAGAGAGTGAGGAAGACGATGAAGGAGATACCGAGGAAGATGATGAAGGAGATAGCGAGGAAGATGATGAAGGAGAGAACAAGGAAGACGAGGATGGAGAAAGCGAGGACTTTGAAGATGGAAATGATAAAGAGAGTGAGAGTGGCGATGAAGGTAATGATGACAATAAAGATGCTCAGATGGAAGAGCTTGAGAAAGAGGTCAAGGAGCTTCGTTCACAAGAGCAgtaagatttcatttgaactaatcatttttaaatactgttatttctaagatagtgaattcttgtatgtgtttctttctagaaacggtagatagtcgtattaaagtttcaactgtttaatctagatcagcaaggatgttggctagatttaCaTATGGCTTTCGATAGAAACGGTGTCAATAGTAGTATTACAACACACCACAGAAAAAGGGACGATCTATTACAGCGTTTCAAAATTGTGGTTTGTTTGATATTAAGCTTCAGACTGAACGTTTCTGGTCTTGAGACCGAAGAGATTCTTAAGCAAATCCGCCGTGTTTGGAGGAATCTTCGCTGGTCGTGGAGCCATAAAGTTGACAATCTTCTCATGAACATTATACCTGTTTCGATTTTTTACAAGAAAATGATTCAAAAGAGAGAATTATGGGAAGAGAAAACCTCCAAGAGGTGAAACTATGTGTGTTTTATTTACCTGATCTTTCTGCTCTTTGAGGCACGTCGATCCACAACTTTCCTCTTCTTCGTCTGGAACTTCTTCATCTCGTAGAAAGCCGCCTCTGCCAATGGCAAATATTGAGATTTCAGAATATAAAAAAGACTCTTTACACTTGAGTTGTTGGACATAACAATCGCTAGGGGGGGTTTCATATCTAATGATTCTAGGATGTTTTTCTTTACTATACCTGAGGAAGCTGGATCGATAGTCTCGAGAAACTCCTTTAGTAACTGCCTATAGAACTCAGCATCTTCCACAAGTTCAGGGTCTCCTTCTTCTTGTTTTTCCTGGACAAGAAAGAGTACACATGTTAGAGAATGGGTATCAAGATAATCAATGAGATTGATTAAATGTTTATACTCTTTATGGTTAGAGAAAACCGTGATTTTACCTCTGGATTTGGTTCCATGGCTTCCTGATATGGCTTTCGATAGAAACGGTGTCAATAGTAGTATTACAACACACCACAGAAAAAGGGACGATCTATTACAGCGTTTCAAAATTGTGGTTTGTTTGATATTAAGCTTCAGACTGAACGTTTCTGGTCTTGAGACCGAAGAGATTCTTAAGCAAATCCGCCGTGTTTGGAGGAATCTTCGCTGGTCGTGGAGCCATAAAGTTGACAATCTTCTCATGAACATTATACCTGTTTCGATTTTTTACAAGAAAATGATTCAAAAGAGAGAATTATGGGAAGAGAAAACCTCCAAGAGGTGAAACTATGTGTGTTTTATTTACCTGATCTTTCTGCTCTTTGAGGCACGTCGATCCACAACTTTCCTCTTCTTCGTCTGGAACTTCTTCATCTCGTAGAAAGCCGCCTCTGCCAATGGCAAATATTGAGATTTCAGAATATAAAAAAGACTCTTTACACTTGAGTTGTTGGACATAACAATCGCTAGGGGGGGTTTCATATCTAATGATTCTAGGATGTTTTTCTTTACTATACCTGAGGAAGCTGGATCGATAGTCTCGAGAAACTCCTTTAGTAACTGCCTATAGAACTCAGCATCTTCCACAAGTTCAGGGTCTCCTTCTTCTTGTTTTTCCTGGACAAGAAAGAGTACACATGTTAGAGAATGGGTATCAAGATAATCAATGAGATTGATTAAATGTTTATACTCTTTATGGTTAGAGAAAACCGTGATTTTACCTCTGGATTTGGTTCCATGGCTTCCTGAGGGACCTGTGGATGTTTagaagtaggttcgaactcattatagcaattggatttattcgtgattagtagaaaatatatacgtttaaaaggaaaagaacagacttttgccaaaagtttttgtcagatactgtcttaattcggttgcatcatttaatttTTTACAAGAAAATGATTCAAAAGAGAGAATTATGGGAAGAGAAAACCTCCAAGAGGTGAAACTATGTGTGTtttaaatgaacgTGTCAATAGTAGTATTACAACACACCACAGAAAAAGGGACGATCTATTACAGCGTTTCAAAATattgagaggcaaaagaatgttctttgtgtaatatgcgtgttcctggtatagGGATATATTGAAGAACTTGAAGCGTGATAAGGGTGAAGATGCTGTTAAAGGTCAAGCGGTGAAGAATCAGAAGgtatgaccttgttctgcggtttgcgaagtagtctaactagaaatcagttgcagttgtatattcctattttatgttaatgaagttgatctagagcgaatttttgacaattctttctttgtgctctatgtaaagGCTCTTTGGGATAAGATTCTGGAGTTCAGATTCTTACTTCAGAAAGCATTTGATCGTTCAAACAGATTACCACAGgtaagctatgctcttctcctcttctGtgGAAGCAACCTGTTCACTAACGTTTtGGTTTtGAAAATTAATTtAAAGTTAGATTCCTGATTGAtCCCCAGAtAAGAGCatttatggttgtttgtttcatcatactttatgacagaaATATATTTTCTACTAATCACGAATAAATCCAATTGCTATAATGAGTTCGAACCTACTTCTAAATCTAGCCAACATCCTTGCTGATCTAGATTAAACAGTTGAAACTTTAATACGACTATCTACCGTTTCTAGAAAGAAACACATACAAGAATTCACTATCTTAGAAATAACAGTATTTAAAAATGATTAGTTCAAATGAAATCTTACTGCTCTTGTGAACGAAGCTCCTTGACCTttTTTACAAGAAAATGATTCAAAAGAGAGAATTATGGGAAGAGAAAACCTCCAAGAGGTGAAACTATGTGTGTTTTATTTACCTGATCTTTCTGCTCTTTGAGGCACGTCGATCCACAACTTTCCTCTTCTTCGTCTGGAACTTCTTCATCTCGTAGAAAGCCGCCTCTGCCAATGGCAAATATTGAGATTTCAGAATATAAAAAAGACTCTTTACACTTGAGTTGTTGGACATAACAATCGCTAGGGGGGGTTTCATATCTAATGATTCTAGGATGTTTTTCTTTACTATACCTGAGGAAGCTGGATCGATAGTCTCGAGAAACTCCTTTAGTAACTGCCTATAGAACTCAGCATCTTCCACAAGTTCAGGGTCTCCTTCTTCTTGTTTTTCCTGGACAAGAAAGAGTACACATGTTAGAGAATGGGTATCAAGATAATCAATGAGATTGATTAAATGTTTATACTCTTTATGGTTAGAGAAAACCGTGATTTTACCTCTGGATTTGGTTCCATGGCTTCCTGAGGGACCTGTGGATGTTTAAATTTAAAGTCAGGAAAAATTGAAGAGAAGCTGATCTATTGACCACCAAAGGCCAAATTCTTAGATGGTTTATGCTTTTATTATCAAAGCAAAAACTTACAGTTCCAAAAACAGCAACAGTAGATCTTGATTGTTGCATCTGTTTAATCATTCTACTTGGATCCCTCATGtgGAAGCAACCTGTTCACTAACGTTTtGGTTTtGAAAATTAATTtAAAGTTAGATTCCTGATTGAtCCCCAGAtAAGAGCCTTTCTCAAGCTCTTCCATCTGAGCATCTTTATTGTCATCATTACCTTCATCGCCACTCTCACTCTCTTTATCATTTCCATCTTCAAAGTCCTCGCTTTCTCCATCCTGACAGATAGAAAAAGAAAGGAgatGtTATATTGACCCTCGCTTATAGTGATGATAATTATGTTATCTGTTAATATGGCATACCATTCACTTGTTGATCAACCGAGGGGTTCTTCTCAAACAAAGCCTGTAGAAATCCATATTTAGATAATAATAATAAAAGTCAACAAACGTGGCAGAGCGACAAAACACTAAGAGACAGCATACAATTACTGAATCAAGCCTATGCATACATACCTCTTGCAACTCCAAGAGGGAATCTAATGTCTTCTTAGATGAAGTAACTAGATCTGTATATGCTGTTGAGACATCCTCATCTTCTGAACAAAATAACGATTTCACAGGCTCCTGAAAAGGGTACCTTCTGTCATAAAGTATGATGAAACAAACAACCATAAATAGAAGAGGAGAAGAGCATAGCTTACCTGTGGTAATCTGTTTGAACGATCAAATGCTTTCTGAAGTAAGAATCTGAACTCCAGAATCTTATCCCAAAGAGCCTTTACATAGAGCACAAAGAAAGAATTGTCAAAAATTCGCTCTAGATCAACTTCATTAACATAAAATAGGAATATACAACTGCAACTGATTTCTAGTTAGACTACTTCGCAAACCGCAGAACAAGGTCATACCTTCTGATTCTTCACCGCTTGACcttCGTCTTCCTTGTTCTCTCCTTCATCATCTTCCTCGCTATCTCCTTCATCATCTTCCTCGGTATCTCCTTCATCGTCTTCCTCACTCTCTCCCTCATCATCTTCCATGCTATCTACTTCATCATCCTCTATCCCATCAGGTAATTGATCATCTTCATTGTCGCTTTCGGCCTATAATATAGAGAACCCTCAACCAAATAAATTCACCTATACCATCTAAATAACTTAAAcgaGacaAAAGCAACAtaACATACCTTaagGTTTTCTTggtCGCTTATGTCTTCCgATTCACTGggtacccttttcagGAGCCTGTGAAATCGTTATTTTGTTCAGAAGATGAGGATGTCTCAACAGCATATACAGATCTAGTTACTTCATCTAAGAAGACATTAGATTCCCTCTTGGAGTTGCAAGAGgtatgtatgcataggcttgattcagtaattgtatgctgtctcttagtgttttgtcgctctgccacgtttgttgacttttattattattatctaaatatggatttctacagGCTTTGTTTGAGAAGAACCCCTCGGTTGATCAACAAGTGAATGgtatgccatattaacagataacataattatcatcactataagcgagggtcaatataacatctcctttctttttctatctgtcagCAACAGCTAGTGAAGAATCCAATAAATCAGATGCAGAAGATAGCGATGAATGGCAGCGAATATCTGACTTGCAGAAAAGgtattcaagactaaatgttcaactgtccagctttaaagttctgtccaatcaaaagaattctgtttagtaaagcatgattgacttcatttggagaccagtctcataaccatttatgtttcatcgttaacacaaatttggtatttgctatgtagAATGTCTGTGTTCCGAAACAAGGCTGTGGACAAATGGCAGAGAAAAACACAAGTCACAACTGGTGCAGCTGCTATTAAAGGAAAGCTCCACGCCTTTAACCAGgtatagaaagcaaactccgaagatgattttgctcttatctggggatcaatcaggaatctaactttaaattaattttcaaaaccagAACGTTAGTGAACAGGTTGCTTCCTACATGAGGGATCCAAGTAGAATGATTAAACAGATGCAACAATCAAGATCTACTGTTGCTGTTTTTGGAACTgtaagtttttgctttgataataaaagcataaaccatctaagaatttggcctttggtggtcaatagatcagcttctcttcaatttttcctgactttaaatttaaacatccacagGTCCCTCAGGAAGCCATGGAACCAAATCCAGAGgtaaaatcacggttttctctaaccataaagagtataaacatttaatcaatctcattgattatcttgatacccattctctaacatgtgtactctttcttgtccagGAAAAACAAGAAGAAGGAGACCCTGAACTTGTGGAAGATGCTGAGTTCTATAGGCAGTTACTAAAGGAGTTTCTCGAGACTATCGATCCAGCTTCCTCAGgtatagtaaagaaaaacatcctagaatcattagatatgaaaccccccctagcgattgttatgtccaacaactcaagtgtaaagagtcttttttatattctgaaatctcaatatttgccattggcagAGGCGGCTTTCTACGAGATGAAGAAGTTCCAGACGAAGAAGAGGAAAGTTGTGGATCGACGTGCCTCAAAGAGCAGAAAGATCAGgtaaataaaacacacatagtttcacctcttggaggttttctcttcccataattctctcttttgaatcattttcttgtaaaaaatcgaaacagGTATAATGTTCATGATCCCTATACCAGGAACACGCATATTACACAAAGAACATTCTTTTGCCGCGAAGATTCCTCCAAACACGGCGGATTTGCTTAAGAATCTCTTCGGTCTCAAGACCAGAAACGTTCAGTCTGAAGCTTAA

In *che1* mutant g changed to a (highlighted with green), which leads to RNA mis-splicing:

ATGGCTGGGGGGTCAAAGAGGTCTAAAAGAGCAAGACTTGACAGTGAATCGGAAGACATAAGCGACCAAGAAAACCTTAAGgtatgtttatgttgcttttgtctcgtttaagttatttagatggtataggtgaatttatttggttgagggttctctatattatagGCCGAAAGCGACAATGAAGATGATCAATTACCTGATGGGATAGAGGATGATGAAGTAGATAGCATGGAAGATGATGAGGGAGAGAGTGAGGAAGACGATGAAGGAGATACCGAGGAAGATGATGAAGGAGATAGCGAGGAAGATGATGAAGGAGAGAACAAGGAAGACGAGGATGGAGAAAGCGAGGACTTTGAAGATGGAAATGATAAAGAGAGTGAGAGTGGCGATGAAGGTAATGATGACAATAAAGATGCTCAGATGGAAGAGCTTGAGAAAGAGGTCAAGGAGCTTCGTTCACAAGAGCAgtaagatttcatttgaactaatcatttttaaatactgttatttctaagatagtgaattcttgtatgtgtttctttctagaaacggtagatagtcgtattaaagtttcaactgtttaatctagatcagcaaggatgttggctagatttaCaTATGGCTTTCGATAGAAACGGTGTCAATAGTAGTATTACAACACACCACAGAAAAAGGGACGATCTATTACAGCGTTTCAAAATTGTGGTTTGTTTGATATTAAGCTTCAGACTGAACGTTTCTGGTCTTGAGACCGAAGAGATTCTTAAGCAAATCCGCCGTGTTTGGAGGAATCTTCGCTGGTCGTGGAGCCATAAAGTTGACAATCTTCTCATGAACATTATACCTGTTTCGATTTTTTACAAGAAAATGATTCAAAAGAGAGAATTATGGGAAGAGAAAACCTCCAAGAGGTGAAACTATGTGTGTTTTATTTACCTGATCTTTCTGCTCTTTGAGGCACGTCGATCCACAACTTTCCTCTTCTTCGTCTGGAACTTCTTCATCTCGTAGAAAGCCGCCTCTGCCAATGGCAAATATTGAGATTTCAGAATATAAAAAAGACTCTTTACACTTGAGTTGTTGGACATAACAATCGCTAGGGGGGGTTTCATATCTAATGATTCTAGGATGTTTTTCTTTACTATACCTGAGGAAGCTGGATCGATAGTCTCGAGAAACTCCTTTAGTAACTGCCTATAGAACTCAGCATCTTCCACAAGTTCAGGGTCTCCTTCTTCTTGTTTTTCCTGGACAAGAAAGAGTACACATGTTAGAGAATGGGTATCAAGATAATCAATGAGATTGATTAAATGTTTATACTCTTTATGGTTAGAGAAAACCGTGATTTTACCTCTGGATTTGGTTCCATGGCTTCCTGATATGGCTTTCGATAGAAACGGTGTCAATAGTAGTATTACAACACACCACAGAAAAAGGGACGATCTATTACAGCGTTTCAAAATTGTGGTTTGTTTGATATTAAGCTTCAGACTGAACGTTTCTGGTCTTGAGACCGAAGAGATTCTTAAGCAAATCCGCCGTGTTTGGAGGAATCTTCGCTGGTCGTGGAGCCATAAAGTTGACAATCTTCTCATGAACATTATACCTGTTTCGATTTTTTACAAGAAAATGATTCAAAAGAGAGAATTATGGGAAGAGAAAACCTCCAAGAGGTGAAACTATGTGTGTTTTATTTACCTGATCTTTCTGCTCTTTGAGGCACGTCGATCCACAACTTTCCTCTTCTTCGTCTGGAACTTCTTCATCTCGTAGAAAGCCGCCTCTGCCAATGGCAAATATTGAGATTTCAGAATATAAAAAAGACTCTTTACACTTGAGTTGTTGGACATAACAATCGCTAGGGGGGGTTTCATATCTAATGATTCTAGGATGTTTTTCTTTACTATACCTGAGGAAGCTGGATCGATAGTCTCGAGAAACTCCTTTAGTAACTGCCTATAGAACTCAGCATCTTCCACAAGTTCAGGGTCTCCTTCTTCTTGTTTTTCCTGGACAAGAAAGAGTACACATGTTAGAGAATGGGTATCAAGATAATCAATGAGATTGATTAAATGTTTATACTCTTTATGGTTAGAGAAAACCGTGATTTTACCTCTGGATTTGGTTCCATGGCTTCCTGAGGGACCTGTGGATGTTTagaagtaggttcgaactcattatagcaattggatttattcgtgattagtagaaaatatatacgtttaaaaggaaaagaacagacttttgccaaaagtttttgtcagatactgtcttaattcggttgcatcatttaatttTTTACAAGAAAATGATTCAAAAGAGAGAATTATGGGAAGAGAAAACCTCCAAGAGGTGAAACTATGTGTGTtttaaatgaacgTGTCAATAGTAGTATTACAACACACCACAGAAAAAGGGACGATCTATTACAGCGTTTCAAAATattgagaggcaaaagaatgttctttgtgtaatatgcgtgttcctggtatagGGATATATTGAAGAACTTGAAGCGTGATAAGGGTGAAGATGCTGTTAAAGGTCAAGCGGTGAAGAATCAGAAGgtatgaccttgttctgcggtttgcgaagtagtctaactagaaatcagttgcagttgtatattcctattttatgttaatgaagttgatctagagcgaatttttgacaattctttctttgtgctctatgtaaagGCTCTTTGGGATAAGATTCTGGAGTTCAGATTCTTACTTCAGAAAGCATTTGATCGTTCAAACAGATTACCACAGgtaagctatgctcttctcctcttctGtgGAAGCAACCTGTTCACTAACGTTTtGGTTTtGAAAATTAATTtAAAGTTAGATTCCTGATTGAtCCCCAGAtAAGAGCatttatggttgtttgtttcatcatactttatgacagaaATATATTTTCTACTAATCACGAATAAATCCAATTGCTATAATGAGTTCGAACCTACTTCTAAATCTAGCCAACATCCTTGCTGATCTAGATTAAACAGTTGAAACTTTAATACGACTATCTACCGTTTCTAGAAAGAAACACATACAAGAATTCACTATCTTAGAAATAACAGTATTTAAAAATGATTAGTTCAAATGAAATCTTACTGCTCTTGTGAACGAAGCTCCTTGACCTttTTTACAAGAAAATGATTCAAAAGAGAGAATTATGGGAAGAGAAAACCTCCAAGAGGTGAAACTATGTGTGTTTTATTTACCTGATCTTTCTGCTCTTTGAGGCACGTCGATCCACAACTTTCCTCTTCTTCGTCTGGAACTTCTTCATCTCGTAGAAAGCCGCCTCTGCCAATGGCAAATATTGAGATTTCAGAATATAAAAAAGACTCTTTACACTTGAGTTGTTGGACATAACAATCGCTAGGGGGGGTTTCATATCTAATGATTCTAGGATGTTTTTCTTTACTATACCTGAGGAAGCTGGATCGATAGTCTCGAGAAACTCCTTTAGTAACTGCCTATAGAACTCAGCATCTTCCACAAGTTCAGGGTCTCCTTCTTCTTGTTTTTCCTGGACAAGAAAGAGTACACATGTTAGAGAATGGGTATCAAGATAATCAATGAGATTGATTAAATGTTTATACTCTTTATGGTTAGAGAAAACCGTGATTTTACCTCTGGATTTGGTTCCATGGCTTCCTGAGGGACCTGTGGATGTTTAAATTTAAAGTCAGGAAAAATTGAAGAGAAGCTGATCTATTGACCACCAAAGGCCAAATTCTTAGATGGTTTATGCTTTTATTATCAAAGCAAAAACTTACAGTTCCAAAAACAGCAACAGTAGATCTTGATTGTTGCATCTGTTTAATCATTCTACTTGGATCCCTCATGtgGAAGCAACCTGTTCACTAACGTTTtGGTTTtGAAAATTAATTtAAAGTTAGATTCCTGATTGAtCCCCAGAtAAGAGCCTTTCTCAAGCTCTTCCATCTGAGCATCTTTATTGTCATCATTACCTTCATCGCCACTCTCACTCTCTTTATCATTTCCATCTTCAAAGTCCTCGCTTTCTCCATCCTGACAGATAGAAAAAGAAAGGAgatGtTATATTGACCCTCGCTTATAGTGATGATAATTATGTTATCTGTTAATATGGCATACCATTCACTTGTTGATCAACCGAGGGGTTCTTCTCAAACAAAGCCTGTAGAAATCCATATTTAGATAATAATAATAAAAGTCAACAAACGTGGCAGAGCGACAAAACACTAAGAGACAGCATACAATTACTGAATCAAGCCTATGCATACATACCTCTTGCAACTCCAAGAGGGAATCTAATGTCTTCTTAGATGAAGTAACTAGATCTGTATATGCTGTTGAGACATCCTCATCTTCTGAACAAAATAACGATTTCACAGGCTCCTGAAAAGGGTACCTTCTGTCATAAAGTATGATGAAACAAACAACCATAAATAGAAGAGGAGAAGAGCATAGCTTACCTGTGGTAATCTGTTTGAACGATCAAATGCTTTCTGAAGTAAGAATCTGAACTCCAGAATCTTATCCCAAAGAGCCTTTACATAGAGCACAAAGAAAGAATTGTCAAAAATTCGCTCTAGATCAACTTCATTAACATAAAATAGGAATATACAACTGCAACTGATTTCTAGTTAGACTACTTCGCAAACCGCAGAACAAGGTCATACCTTCTGATTCTTCACCGCTTGACcttCGTCTTCCTTGTTCTCTCCTTCATCATCTTCCTCGCTATCTCCTTCATCATCTTCCTCGGTATCTCCTTCATCGTCTTCCTCACTCTCTCCCTCATCATCTTCCATGCTATCTACTTCATCATCCTCTATCCCATCAGGTAATTGATCATCTTCATTGTCGCTTTCGGCCTATAATATAGAGAACCCTCAACCAAATAAATTCACCTATACCATCTAAATAACTTAAAcgaGacaAAAGCAACAtaACATACCTTaagGTTTTCTTggtCGCTTATGTCTTCCgATTCACTGggtacccttttcagGAGCCTGTGAAATCGTTATTTTGTTCAGAAGATGAGGATGTCTCAACAGCATATACAGATCTAGTTACTTCATCTAAGAAGACATTAGATTCCCTCTTGGAGTTGCAAGAGgtatgtatgcataggcttgattcagtaattgtatgctgtctcttagtgttttgtcgctctgccacgtttgttgacttttattattattatctaaatatggatttctacagGCTTTGTTTGAGAAGAACCCCTCGGTTGATCAACAAGTGAATGgtatgccatattaacagataacataattatcatcactataagcgagggtcaatataacatctcctttctttttctatctgtcagCAACAGCTAGTGAAGAATCCAATAAATCAGATGCAGAAGATAGCGATGAATGGCAGCGAATATCTGACTTGCAGAAAAGgtattcaagactaaatgttcaactgtccagctttaaagttctgtccaatcaaaagaattctgtttagtaaagcatgattgacttcatttggagaccagtctcataaccatttatgtttcatcgttaacacaaatttggtatttgctatgtagAATGTCTGTGTTCCGAAACAAGGCTGTGGACAAATGGCAGAGAAAAACACAAGTCACAACTGGTGCAGCTGCTATTAAAGGAAAGCTCCACGCCTTTAACCAGgtatagaaagcaaactccgaagatgattttgctcttatctggggatcaatcaggaatctaactttaaattaattttcaaaaccaaAACGTTAGTGAACAGGTTGCTTCCTACATGAGGGATCCAAGTAGAATGATTAAACAGATGCAACAATCAAGATCTACTGTTGCTGTTTTTGGAACTgtaagtttttgctttgataataaaagcataaaccatctaagaatttggcctttggtggtcaatagatcagcttctcttcaatttttcctgactttaaatttaaacatccacagGTCCCTCAGGAAGCCATGGAACCAAATCCAGAGgtaaaatcacggttttctctaaccataaagagtataaacatttaatcaatctcattgattatcttgatacccattctctaacatgtgtactctttcttgtccagGAAAAACAAGAAGAAGGAGACCCTGAACTTGTGGAAGATGCTGAGTTCTATAGGCAGTTACTAAAGGAGTTTCTCGAGACTATCGATCCAGCTTCCTCAGgtatagtaaagaaaaacatcctagaatcattagatatgaaaccccccctagcgattgttatgtccaacaactcaagtgtaaagagtcttttttatattctgaaatctcaatatttgccattggcagAGGCGGCTTTCTACGAGATGAAGAAGTTCCAGACGAAGAAGAGGAAAGTTGTGGATCGACGTGCCTCAAAGAGCAGAAAGATCAGgtaaataaaacacacatagtttcacctcttggaggttttctcttcccataattctctcttttgaatcattttcttgtaaaaaatcgaaacagGTATAATGTTCATGATCCCTATACCAGGAACACGCATATTACACAAAGAACATTCTTTTGCCGCGAAGATTCCTCCAAACACGGCGGATTTGCTTAAGAATCTCTTCGGTCTCAAGACCAGAAACGTTCAGTCTGAAGCTTAA

*AtCHE1* wild type (AT5G61330) CDS:

ATGGCTGGGGGGTCAAAGAGGTCTAAAAGAGCAAGACTTGACAGTGAATCGGAAGACATAAGCGACCAAGAAAACCTTAAGGCCGAAAGCGACAATGAAGATGATCAATTACCTGATGGGATAGAGGATGATGAAGTAGATAGCATGGAAGATGATGAGGGAGAGAGTGAGGAAGACGATGAAGGAGATACCGAGGAAGATGATGAAGGAGATAGCGAGGAAGATGATGAAGGAGAGAACAAGGAAGACGAGGATGGAGAAAGCGAGGACTTTGAAGATGGAAATGATAAAGAGAGTGAGAGTGGCGATGAAGGTAATGATGACAATAAAGATGCTCAGATGGAAGAGCTTGAGAAAGAGGTCAAGGAGCTTCGTTCACAAGAGCAGGATATATTGAAGAACTTGAAGCGTGATAAGGGTGAAGATGCTGTTAAAGGTCAAGCGGTGAAGAATCAGAAGGCTCTTTGGGATAAGATTCTGGAGTTCAGATTCTTACTTCAGAAAGCATTTGATCGTTCAAACAGATTACCACAGGAGCCTGTGAAATCGTTATTTTGTTCAGAAGATGAGGATGTCTCAACAGCATATACAGATCTAGTTACTTCATCTAAGAAGACATTAGATTCCCTCTTGGAGTTGCAAGAGGCTTTGTTTGAGAAGAACCCCTCGGTTGATCAACAAGTGAATGCAACAGCTAGTGAAGAATCCAATAAATCAGATGCAGAAGATAGCGATGAATGGCAGCGAATATCTGACTTGCAGAAAAGAATGTCTGTGTTCCGAAACAAGGCTGTGGACAAATGGCAGAGAAAAACACAAGTCACAACTGGTGCAGCTGCTATTAAAGGAAAGCTCCACGCCTTTAACCAG***AACGTTAG***TGAACAGGTTGCTTCCTACATGAGGGATCCAAGTAGAATGATTAAACAGATGCAACAATCAAGATCTACTGTTGCTGTTTTTGGAACTGTCCCTCAGGAAGCCATGGAACCAAATCCAGAGGAAAAACAAGAAGAAGGAGACCCTGAACTTGTGGAAGATGCTGAGTTCTATAGGCAGTTACTAAAGGAGTTTCTCGAGACTATCGATCCAGCTTCCTCAGAGGCGGCTTTCTACGAGATGAAGAAGTTCCAGACGAAGAAGAGGAAAGTTGTGGATCGACGTGCCTCAAAGAGCAGAAAGATCAGGTATAATGTTCATGAGAAGATTGTCAACTTTATGGCTCCACGACCAGCGAAGATTCCTCCAAACACGGCGGATTTGCTTAAGAATCTCTTCGGTCTCAAGACCAGAAACGTTCAGTCTGAAGCTTAA

In *che1* mutant CDS, the yellow highlighted fragment in *CHE1* wild type ‘AACGTTAG’ is missing.

ATGGCTGGGGGGTCAAAGAGGTCTAAAAGAGCAAGACTTGACAGTGAATCGGAAGACATAAGCGACCAAGAAAACCTTAAGGCCGAAAGCGACAATGAAGATGATCAATTACCTGATGGGATAGAGGATGATGAAGTAGATAGCATGGAAGATGATGAGGGAGAGAGTGAGGAAGACGATGAAGGAGATACCGAGGAAGATGATGAAGGAGATAGCGAGGAAGATGATGAAGGAGAGAACAAGGAAGACGAGGATGGAGAAAGCGAGGACTTTGAAGATGGAAATGATAAAGAGAGTGAGAGTGGCGATGAAGGTAATGATGACAATAAAGATGCTCAGATGGAAGAGCTTGAGAAAGAGGTCAAGGAGCTTCGTTCACAAGAGCAGGATATATTGAAGAACTTGAAGCGTGATAAGGGTGAAGATGCTGTTAAAGGTCAAGCGGTGAAGAATCAGAAGGCTCTTTGGGATAAGATTCTGGAGTTCAGATTCTTACTTCAGAAAGCATTTGATCGTTCAAACAGATTACCACAGGAGCCTGTGAAATCGTTATTTTGTTCAGAAGATGAGGATGTCTCAACAGCATATACAGATCTAGTTACTTCATCTAAGAAGACATTAGATTCCCTCTTGGAGTTGCAAGAGGCTTTGTTTGAGAAGAACCCCTCGGTTGATCAACAAGTGAATGCAACAGCTAGTGAAGAATCCAATAAATCAGATGCAGAAGATAGCGATGAATGGCAGCGAATATCTGACTTGCAGAAAAGAATGTCTGTGTTCCGAAACAAGGCTGTGGACAAATGGCAGAGAAAAACACAAGTCACAACTGGTGCAGCTGCTATTAAAGGAAAGCTCCACGCCTTTAACCAGTGAACAGGTTGCTTCCTACATGAGGGATCCAAGTAGAATGATTAAACAGATGCAACAATCAAGATCTACTGTTGCTGTTTTTGGAACTGTCCCTCAGGAAGCCATGGAACCAAATCCAGAGGAAAAACAAGAAGAAGGAGACCCTGAACTTGTGGAAGATGCTGAGTTCTATAGGCAGTTACTAAAGGAGTTTCTCGAGACTATCGATCCAGCTTCCTCAGAGGCGGCTTTCTACGAGATGAAGAAGTTCCAGACGAAGAAGAGGAAAGTTGTGGATCGACGTGCCTCAAAGAGCAGAAAGATCAGGTATAATGTTCATGAGAAGATTGTCAACTTTATGGCTCCACGACCAGCGAAGATTCCTCCAAACACGGCGGATTTGCTTAAGAATCTCTTCGGTCTCAAGACCAGAAACGTTCAGTCTGAAGCTTAA
