# Supplementary material for: AtCHE1, the Arabidopsis homolog of mammalian AATF/Che-1 protein, is involved in safeguarding genome stability
Source: Commun Biol. 2025 Sep 2;8:1329. doi: 10.1038/s42003-025-08490-1 (PMC12402150; doi:10.1038/s42003-025-08490-1)
Supplement: Supplementary file 6 — nr-reporting-summary [file 42003_2025_8490_MOESM6_ESM.pdf]

nature portfolio

Corresponding author(s): Klaus Palme

Last updated by author(s): May 25, 2025



# Reporting Summary

Nature Portfolio wishes to improve the reproducibility of the work that we publish. This form provides structure for consistency and transparency in reporting. For further information on Nature Portfolio policies, see our Editorial Policies and the Editorial Policy Checklist.

## Statistics

For all statistical analyses, confirm that the following items are present in the figure legend, table legend, main text, or Methods section.

| n/a | Confirmed | |
|---|---|---|
| ☐ | ☒ | The exact sample size (*n*) for each experimental group/condition, given as a discrete number and unit of measurement |
| ☐ | ☒ | A statement on whether measurements were taken from distinct samples or whether the same sample was measured repeatedly |
| ☐ | ☒ | The statistical test(s) used AND whether they are one- or two-sided<br>*Only common tests should be described solely by name; describe more complex techniques in the Methods section.* |
| ☒ | ☐ | A description of all covariates tested |
| ☐ | ☒ | A description of any assumptions or corrections, such as tests of normality and adjustment for multiple comparisons |
| ☐ | ☒ | A full description of the statistical parameters including central tendency (e.g. means) or other basic estimates (e.g. regression coefficient) AND variation (e.g. standard deviation) or associated estimates of uncertainty (e.g. confidence intervals) |
| ☐ | ☒ | For null hypothesis testing, the test statistic (e.g. *F*, *t*, *r*) with confidence intervals, effect sizes, degrees of freedom and *P* value noted<br>*Give P values as exact values whenever suitable.* |
| ☒ | ☐ | For Bayesian analysis, information on the choice of priors and Markov chain Monte Carlo settings |
| ☒ | ☐ | For hierarchical and complex designs, identification of the appropriate level for tests and full reporting of outcomes |
| ☒ | ☐ | Estimates of effect sizes (e.g. Cohen's *d*, Pearson's *r*), indicating how they were calculated |

*Our web collection on statistics for biologists contains articles on many of the points above.*

## Software and code

Policy information about availability of computer code

| | |
|---|---|
| Data collection | NLS mapper (http://nls-mapper.iab.keio.ac.jp). Nucleolar localization sequence is detected by NoD: NuclOar localization sequence Detector(dundee.ac.uk) (htto:/www.compbio.dundee.ac.uk/www-nod/index.isp). The phosphorylation site of AtCHE1 was predicted by the NetPhos 3.1 server (http:/www.cbs.dtu.dk/services/Netphos/) (Protein domain structure was generated by DOG. Visualization of protein domain structure version 2.0.<br>For images processing and exposing, ZEN lite (Carl Zeiss Microimaing, NIS-Elements Viewer (Nikon), Image J software (National institutes of Health; http://rsb.info.nih.gov/ij) and Fiji were used.<br>For comet assay, Comet analysis was performed using the TriTek Comet Score software.<br>For alignment of the AATF/che-1 homologs, clustalw2 (http://www.ebi.ac.uk/Tools/msa/clustalw2/) was used<br>For phylogenetic analysis of AATF/Che-1 homologs, Mega5.05, align by clustal W was used. |
| Data analysis | For statistical analysis: IBM SPSS statistics 21 and Microsoft Excel |

For manuscripts utilizing custom algorithms or software that are central to the research but not yet described in published literature, software must be made available to editors and reviewers. We strongly encourage code deposition in a community repository (e.g. GitHub). See the Nature Portfolio guidelines for submitting code & software for further information.

## Data

Policy information about availability of data

All manuscripts must include a data availability statement. This statement should provide the following information, where applicable:

- Accession codes, unique identifiers, or web links for publicly available datasets
- A description of any restrictions on data availability
- For clinical datasets or third party data, please ensure that the statement adheres to our policy

The original data that support the findings of this study can be obtained from the corresponding author upon request.

## Research involving human participants, their data, or biological material

Policy information about studies with human participants or human data. See also policy information about sex, gender (identity/presentation), and sexual orientation and race, ethnicity and racism.

| | |
|---|---|
| Reporting on sex and gender | n/a |
| Reporting on race, ethnicity, or other socially relevant groupings | n/a |
| Population characteristics | n/a |
| Recruitment | n/a |
| Ethics oversight | n/a |

Note that full information on the approval of the study protocol must also be provided in the manuscript.

# Field-specific reporting

Please select the one below that is the best fit for your research. If you are not sure, read the appropriate sections before making your selection.

☒ Life sciences ☐ Behavioural & social sciences ☐ Ecological, evolutionary & environmental sciences

For a reference copy of the document with all sections, see nature.com/documents/nr-reporting-summary-flat.pdf

# Life sciences study design

All studies must disclose on these points even when the disclosure is negative.

| | |
|---|---|
| Sample size | One mutant line was used for this analysis. For phenotype analysis, at least 10 seedlings were used. For qPCR, data were from three independent biological repeat. |
| Data exclusions | Arabidopsis seedlings for measurement with failures in treatment applicable and extreme damage after treatment were excluded from analysis. |
| Replication | Depending on the difference purpose, some experiments were repeat at least three times. And the replication times were indicated in the figure legends. |
| Randomization | Random plants were picked for measurement except the exclusions. |
| Blinding | No blinding was used. Because measurements were not vulnerable to observer bias. |

# Reporting for specific materials, systems and methods

We require information from authors about some types of materials, experimental systems and methods used in many studies. Here, indicate whether each material, system or method listed is relevant to your study. If you are not sure if a list item applies to your research, read the appropriate section before selecting a response.

## Materials & experimental systems

| n/a | Involved in the study | |
|---|---|---|
| ☐ | ☒ | Antibodies |
| ☒ | ☐ | Eukaryotic cell lines |
| ☒ | ☐ | Palaeontology and archaeology |
| ☒ | ☐ | Animals and other organisms |
| ☒ | ☐ | Clinical data |
| ☒ | ☐ | Dual use research of concern |
| ☐ | ☒ | Plants |

## Methods

| n/a | Involved in the study | |
|---|---|---|
| ☒ | ☐ | ChIP-seq |
| ☒ | ☐ | Flow cytometry |
| ☒ | ☐ | MRI-based neuroimaging |

# Antibodies

Antibodies used

A rabbit ant-plant yH2AX antibody. A goat anti-rabbit lgG (H+L) high Cross-Adsorbed secondary antibody, Alexa Fluor 488 (invitrogen, A-11034)

Validation

The reference for rabbit anti-plant yH2AX antibody: Amiard, S., C. Charbonnel, E. ALLAlN, A. Depeiges, C. White and M. E. GALLEGO (2010). "Distinct roles of the ATR kinase and the Mre11-Rad51-Nbs1 complex in the maintenance of chromosomal stability in Arabidopsis." Plant Cell 22(9):3020-3033.
The website for goat anti-rabbit lgG (H+L) high Cross-Adsorbed secondary antibody, Alexa fluor 488: https://www.thermofisher.cn/cn/zh/antibody/product/Goat-anti-Rabbit-lgG-H-L-Highly-Cross-Adsorbed-Secondary-Antibody-Polyclonal/A-11034

# Plants

Seed stocks

Arabidopsis seeds used in our study are detaild in the materials and methods.

Novel plant genotypes

A mutant from EMS mutagenesis on an Arabidosis population (ecotype: Col-0). Genomic DNA and coding sequence information for mutant were provided in supplementary data 1.

Authentication

Genotypes can be distinguised through PCR primers in the supplementary table 1
